# Providing an *in vitro* depiction of microglial cells challenged with immunostimulatory extracellular vesicles of *Naegleria fowleri*

**DOI:** 10.3389/fmicb.2024.1346021

**Published:** 2024-02-05

**Authors:** Lissette Retana Moreira, Alberto Cornet-Gomez, M. Rosario Sepulveda, Silvia Molina-Castro, Johan Alvarado-Ocampo, Frida Chaves Monge, Mariana Jara Rojas, Antonio Osuna, Elizabeth Abrahams Sandí

**Affiliations:** ^1^Departamento de Parasitología, Facultad de Microbiología, Universidad de Costa Rica, San José, Costa Rica; ^2^Centro de Investigación en Enfermedades Tropicales (CIET), Universidad de Costa Rica, San José, Costa Rica; ^3^Grupo de Bioquímica y Parasitología Molecular (CTS 183), Departamento de Parasitología, Campus de Fuentenueva, Instituto de Biotecnología, Universidad de Granada, Granada, Spain; ^4^Departamento de Biología Celular, Facultad de Ciencias, Universidad de Granada, Granada, Spain; ^5^Instituto de Investigaciones en Salud (INISA), Universidad de Costa Rica, San José, Costa Rica; ^6^Departamento de Bioquímica, Escuela de Medicina, Universidad de Costa Rica, San José, Costa Rica

**Keywords:** extracellular vesicles, trophozoites, *Naegleria fowleri*, microglia, cytokines, morphological changes, DNA

## Abstract

*Naegleria fowleri* is the causative agent of primary amoebic meningoencephalitis, a rapid and acute infection of the central nervous system with a fatal outcome in >97% of cases. Due to the infrequent report of cases and diagnostic gaps that hinder the possibility of recovering clinic isolates, studies related to pathogenesis of the disease are scarce. However, the secretion of cytolytic molecules has been proposed as a factor involved in the progression of the infection. Several of these molecules could be included in extracellular vesicles (EVs), making them potential virulence factors and even modulators of the immune response in this infection. In this work, we evaluated the immunomodulatory effect of EVs secreted by two clinic isolates of *Naegleria fowleri* using *in vitro* models. For this purpose, characterization analyses between EVs produced by both isolates were first performed, for subsequent gene transcription analyses post incubation of these vesicles with primary cultures from mouse cell microglia and BV-2 cells. Analyses of morphological changes induced in primary culture microglia cells by the vesicles were also included, as well as the determination of the presence of nucleic acids of *N. fowleri* in the EV fractions. Results revealed increased expression of *NOS,* proinflammatory cytokines *IL-6*, *TNF-α*, and *IL-23*, and the regulatory cytokine *IL-10* in primary cultures of microglia, as well as increased expression of *NOS* and *IL-13* in BV-2 cells. Morphologic changes from homeostatic microglia, with small cellular body and long processes to a more amoeboid morphology were also observed after the incubation of these cells with EVs. Regarding the presence of nucleic acids, specific *Naegleria fowleri* DNA that could be amplified using both conventional and qPCR was confirmed in the EV fractions. Altogether, these results confirm the immunomodulatory effects of EVs of *Naegleria fowleri* over microglial cells and suggest a potential role of these vesicles as biomarkers of primary acute meningoencephalitis.

## 1 Introduction

Primary amoebic meningoencephalitis (PAM) is an infection of the central nervous system (CNS) produced by *Naegleria fowleri*, a thermophilic free-living amoeba (FLA) that can be found in different water sources and in soil. This infection is characterized by an acute and fulminant course, with initial symptoms that are undistinguishable from bacterial meningitis, a fact that complicates its diagnosis. Since the first description of the infection (Fowler and Carter, 1965), approximately 440 cases have been reported worldwide (Jahangeer et al., 2020); around one third of these cases have occurred in the Unites States, a country in which is suggested an underestimation of cases that could exceed 50% (Matanock et al., 2018). Due to the low number of diagnosed cases, PAM is considered a rare disease; however, mortality rates of this disease surpass 97% (CDC, 2023). The most affected population includes children and young adults and among the risk factors, aquatic activities like diving, waterskiing, surfing, swimming, exposition to hot springs, and nasal rinsing with tap water can be listed, as *N. fowleri* enters the host through the nose (Pana et al., 2023).

Since more than two decades, the study of free-living amoebae and its impact in human health has increased. In this sense, research has focused in trying to identify the mechanisms employed by these organisms to produce damage, as well as possible diagnostic and therapeutic alternatives for this type of infections. For *N. fowleri*, some virulence factors that could contribute to the pathogenesis of the infection have been identified and can be classified into contact-dependent and contact-independent. It has been also demonstrated that the adhesion of trophozoites of the amoeba to the nasal mucosa via integrin-like adhesins and fibronectin binding protein is a critical initial step during the infection process. Once the amoeba is adhered to the mucosa, the increase in its locomotion rate and the chemotactic response to components of the CNS are crucial factors for the progression of the disease (Marciano-Cabral and Cabral, 2007; Naqvi et al., 2016). Moreover, food cups employed by the amoeba for trogocytosis and the secretion of cytolytic molecules like neuraminidases, hydrolases, phospholipases and pore-forming proteins (naegleriapores A and B) participate in the invasion process and in tissue damage.

The invasion of *Naegleria fowleri* to the host induces an intense immune response, characterized by the activation of innate defense mechanisms during the early stages of the infection, including an increased secretion of mucin (MUC5AC) and the production of IL-8 and IL-1β by respiratory epithelial cells (Siddiqui et al., 2016). Once the amoeba reaches the brain, an intense inflammatory response is produced, characterized by tissue infiltration of eosinophils, neutrophils, and macrophages, as well as increased levels of TNF-α, which is considered to stimulate the adherence of neutrophils to the amoeba, triggering its destruction (Marciano-Cabral and Cabral, 2007).

It has been proposed that activated macrophages have an amoebicidal effect over *N. fowleri* by the production of reactive oxygen species (ROS) during the oxidative burst, besides nitric oxide (NO) and mediators like TNF-α and IL-1 (Siddiqui et al., 2016). Studies performed by other groups using microglia, the primary immune cells in the brain, confirm the production of inflammatory cytokines after the contact with trophozoites of the amoeba, reporting robust levels of mRNAs for *IL-1α*, *IL-1β*, *IL-6*, and *TNF-α* after 6 h of incubation (Marciano-Cabral et al., 2001; Marciano-Cabral and Cabral, 2007). Regarding the role of the immune response during this infection, recent investigations have focused in analyzing excretion/secretion products of the amoeba as possible modulators of this response in the host. Within the secreted products by different microorganisms and its target cells, research in extracellular vesicles (EV) has played a leading role in this and other protozoan microorganisms (Wan et al., 2022).

Initially considered as cellular waste products, the role of EVs in intercellular communication is now fully recognized, a process that is highly conserved among eukaryotic and prokaryotic cells. Moreover, the participation of EVs in inflammatory processes and the transference of genetic information has been confirmed, also demonstrating that their cargo, as well as structural molecules of the vesicles, could trigger and modulate the immune response (Islek et al., 2022). For example, in the case of *Plasmodium,* it has been reported that EVs are able to regulate the immune activity, increasing the parasite’s survival inside the host and considering these vesicles key for the pathogenesis of the disease (Opadokun and Rohrbach, 2021). For extracellular protozoan parasites like *Entamoeba histolytica*, EVs have shown a role in NETosis and ROS production by neutrophils (Díaz-Godínez et al., 2022), while exosomes of *Trichomonas vaginalis* can induce IL-6 production and downregulate the expression of IL-8 (cytokine that recruits neutrophils) in cells of the vaginal epithelium; besides, it has been shown that EVs could modulate the immune response of macrophages *in vitro,* stimulating the release of NO and inducing IL-10 production (Nievas et al., 2020).

Regarding FLA, the production and characterization of EVs has been recently documented. For *Acanthamoeba,* biological and nanomechanical properties of EVs secreted by clinical and environmental isolates have been reported (de Souza Gonçalves et al., 2018; Gonçalves et al., 2019; Retana Moreira et al., 2020a), including an analysis of the immunostimulatory effect of these vesicles over the THP-1 cell line that revealed an increment in transcription levels of cytokines IL-6 and IL-12 (Lin et al., 2019). For *N. fowleri*, the isolation and characterization of EVs from two different clinic isolates was achieved in 2022, demonstrating the presence of proteins as part of their cargo, as well as the induction of the expression of costimulatory molecules and IL-8 in THP-1 macrophages (Lertjuthaporn et al., 2022; Retana Moreira et al., 2022). Taking into account that brain tissue is the target of *N. fowleri* and that the immune response contributes significantly to the damage produced during the infection with this species, the aim of the present work is to evaluate the effect of EVs secreted by two clinic isolates of *N. fowleri* using an *in vitro* model with primary cultures of mouse brain microglia and BV-2 cells, a microglial cell line derived from C57/BL6 mice. For this purpose, transcription levels of different cytokines and *NOS* after the incubation of these cells with EVs secreted by trophozoites of the amoeba during different time points were determined. Characterization analysis of EVs secreted by both *N. fowleri* isolates, analyses of morphological changes induced by these vesicles in primary cultures of mouse brain microglia and a preliminary detection of nucleic acids (including bioactive DNA) of *N. fowleri* in the EV fractions were also achieved.

## 2 Materials and methods

### 2.1 Axenic culture of *Naegleria fowleri* trophozoites

Trophozoites of two clinic isolates of *Naegleria fowleri* from Costa Rica (accession numbers MT090627 and MT210902) (Retana Moreira et al., 2020b) were cultured in 75 cm^2^ Nunc EasYFlask cell culture flasks (Thermo Fisher Scientific, Waltham, Massachusetts, United States) with 2% casein hydrolysate (Sigma Aldrich, Missouri, United States) culture medium, supplemented with 10% inactivated fetal bovine serum (Gibco, GranIsland, New York, United States) and antibiotics (penicillin/streptomycin). The flasks were incubated at 37°C, with daily observation of the cultures under an inverted microscope. For each flask, the culture medium was replaced, at least, every 2 days.

### 2.2 Animal handling and permission of the animal welfare and ethics committee

The experiments performed using animals were approved by the Ethical Committee of the University of Granada (235-CEEA-OH-2018) and by the authorities of the Regional Government of Andalucía (JJAA) (number 12/11/2017/162). The use of animals was performed according to the institutional guidelines (Spanish government regulations) (Real Decreto RD1201/05) and the guidelines of the European Union (European Directive 2010/63/EU).

### 2.3 Isolation of extracellular vesicles

Extracellular vesicles of trophozoites of each *Naegleria fowleri* isolate were obtained as previously described by Retana Moreira et al. (2022), following the Minimal Information for the Study of Extracellular Vesicles (MISEV) guidelines (Théry et al., 2018). Briefly, trophozoites were washed 3 times using sterile PBS and then, 5 × 10^7^ trophozoites were incubated for 5 h at 37°C in 75 cm^2^ Nunc EasYFlask cell culture flasks (Thermo Fisher Scientific, Waltham, Massachusetts, United States) with 3.5 mL of 2% casein hydrolysate (Sigma Aldrich, Missouri, United States) culture medium without serum nor antibiotics. After this incubation, the supernatants were collected and centrifuged at 3,500 × g for 15 min at 4°C to remove possible remaining trophozoites. The resulting supernatants were collected again for extracellular vesicle purification as previously described (Retana Moreira et al., 2022), applying a protocol that includes a centrifugation step at 16,000 × g for 30 min at 4°C to remove larger vesicles, filtration of the supernatant using 0.22 μm pore filters (Sartorius, Göttingen, Germany) and ultracentrifugation steps at 120,000 × g for 150 min at 4°C in a Sorwall^™^ WX80 ultracentrifuge (Thermo Fisher Scientific, Waltham, Massachusetts, United States). The resulting pellets were washed two times in sterile filtered (0.22 μm pore filter) PBS at 120,000 × g for 150 min and suspended in 100 μL sterile PBS. The viability of trophozoites after the 5 h secretion period was evaluated using the trypan blue exclusion test and the protein concentration of each sample was quantified using the Micro-BCA protein assay (Thermo Fischer Scientific, Waltham, Massachusetts, United States), following the manufacturer’s instructions.

The characterization of EVs secreted by each isolate was performed using transmission electron microscopy (TEM), scanning electron microscopy (SEM) and nanoparticle tracking analysis (NTA), as described in previous works (Retana Moreira et al., 2019, 2021, 2022; Cornet-Gomez et al., 2023).

### 2.4 Transmission electron microscopy

To confirm the production of extracellular vesicles by each *Naegleria fowleri* isolate, pellets of the samples obtained after the ultracentrifugation steps were fixed in 500 μL of Karnovsky’s fixative (2.5% glutaraldehyde and 2% formaldehyde in 0.1 M cacodylate buffer, 50 mg of CaCl_2_ in 100 mL) for 2 h at 37°C. Then, the samples were dehydrated and embedded in Spurr resin (Sigma Aldrich, Missouri, United States) and ultra-thin sections were performed and stained using 1% uranyl acetate. Final examination of the samples was performed using a Carl Zeiss LIBRA 120 PLUS SMT electron microscope (Carl Zeiss, Oberkochen, Germany).

### 2.5 Scanning electron microscopy

Trophozoites of each isolate of *Naegleria fowleri* were washed 3 times using sterile PBS and 5 × 10^4^ trophozoites were suspended in 2% casein hydrolysate (Sigma Aldrich, Missouri, United States) culture medium and placed in 18 mm round coverslips (Fisher Scientific, New Hampshire, United States). After 5 h of incubation at 37°C, the coverslips with trophozoites were carefully fixed with 2.5% glutaraldehyde in cacodylate buffer with 0.1 M saccharose and maintained in the fixative solution for 24 h at 4°C. Then, the samples were dehydrated in a graded series of ethanol, desiccated using a critical point dryer (Leica EM CPD 300) and then evaporated with high vacuum carbon coater (Emitech K975X) as described by Díaz Lozano et al. (2017). The samples were finally carbon-coated for 3 min and analyzed using a Zeiss Supra 40VP high-resolution scanning electron microscope.

### 2.6 Nanoparticle tracking analysis

Distribution, size, and concentration of extracellular vesicles of *Naegleria fowleri* were determined by measuring the rate of Brownian motion according to the particle size, using a Nanosight NS300 (Malvern Panalytical, Worcestershire, UK). The system was equipped with a sCMOS camera and a blue 488 nm laser beam.

Before the analysis, the samples were diluted 1/100 in low-binding Eppendorf tubes with sterile-filtered (0.22 μm pore filter) PBS. Measurements were performed at 25°C. For data acquisition and information processing, the NTA software 3.2 Dev Build 3.2.16 was employed.

### 2.7 Protein pattern and recognition of extracellular vesicles secreted by each isolate by polyclonal anti-*Naegleria fowleri* antibodies

#### 2.7.1 Preparation of whole protein extracts of *Naegleria fowleri* trophozoites

Whole protein extracts of lysates of each isolate of *Naegleria fowleri* were obtained as previously described (Retana Moreira et al., 2022). Briefly, 5 × 10^7^ trophozoites were washed three times in sterile PBS, suspended in 500 μL sterile PBS and submitted to sonication in a 4710 series ultrasonic homogenizer (Cole-Parmer Instrument Co., Illinois, United States) applying 3 cycles of 30 s, with a 60 s pause between cycles. Protein quantification of the whole protein extracts was also achieved using the Micro-BCA protein assay (Thermo Fischer Scientific, Waltham, Massachusetts, United States).

#### 2.7.2 Electrophoretic separation of proteins using SDS-PAGE

To obtain protein profiles, samples of extracellular vesicles and whole protein extracts of each isolate of *Naegleria fowleri* were diluted 1:1 in sample buffer (Laemmli, 1970), heated for 10 min at 98°C and subsequently loaded onto 12% SDS-polyacrylamide gels. Electrophoretic runs were performed for 90 min (120 V). Once the electrophoresis was completed, silver stain was performed, following previously described protocols (Heukeshoven and Dernick, 1988).

#### 2.7.3 Polyclonal antibody production

Polyclonal anti-*Naegleria fowleri* antibodies were produced after the immunization of four-week-old female Wistar rats with 40 μg of whole protein extract of lysates of trophozoites of *Naegleria fowleri* (ATCC *N. fowleri* Carter 30808), following the methodology previously described by our group (Retana Moreira et al., 2022). Briefly, the antigen was prepared by emulsification of the amoebae lysate (prepared in sterile PSB) in complete Freund’s adjuvant (Sigma, Ronkonkoma, NY, United States), using a 1:1 ratio (final volume: 500 μL). This emulsion was administered intraperitoneally to the rats. For subsequent immunizations, the adjuvant was switched to incomplete Freund’s adjuvant (Sigma-Aldrich, St. Louis, MO, United States). A total of 8 immunizations (1 per week) were performed and the antibody production was evaluated using ELISA and Western blot (WB), as described elsewhere (Towbin et al., 1979).

#### 2.7.4 Western blot

The recognition of the origin of extracellular vesicles, as coming from trophozoites of *Naegleria fowleri,* was performed by Western blot, where proteins separated from lysates of trophozoites and EVs using SDS-PAGE electroforesis were confronted to polyclonal anti-*N. fowleri* antibodies obtained as described above. Briefly, SDS-PAGE electrophoresis was performed, the separated proteins in the polyacrylamide gels were transferred to nitrocellulose membranes (60 min, 90 V) in an Enduro VE10 Vertical Gel System (Labnet International, New Jersey, United States) and, after the transference, the membranes were blocked overnight with 5% non-fat milk in PBS-0.1% Tween 20, washed four times in a solution of PBS-0.1% Tween 20 and incubated overnight at 4°C with the polyclonal anti-*Naegleria fowleri* antibodies (1: 10,000). After the incubation, the membranes were washed and incubated for 1 h with peroxidase-conjugated goat anti-rat IgGs (1, 10,000) (Thermo Scientific, Massachussetts, United States) and, once four washing steps with PBS-0.1% Tween 20 were performed, the reaction was visualized using the Clarity ECL Western substrate (BioRad, California, United States) in a ChemiDoc Imaging system (BioRad, California, United States).

### 2.8 Cytokine expression analyses in microglial cultures

#### 2.8.1 Primary culture from mouse cell microglia and BV-2 cell line culture

Primary cultures of mouse brain microglia were prepared according to Morales-Ropero et al. (2021). Briefly, newborn (1-day old) C57BL/6 mice were obtained from the animal facility service of the “Centro de Instrumentación Científica” at the University of Granada (UGR) and meninges-free cerebral cortex from the brains were dissected and collected in DMEM with 4.5 g/L D-glucose, 4 mM glutamine, 10% fetal bovine serum, 10% horse serum, 100 U/mL penicillin and 100 μg/mL streptomycin (all reagents from GIBCO, Waltham, Massachusetts, United States). After disaggregation and homogenization, cells were seeded and incubated at 37°C with 5% CO_2_ for 10–12 days. Then, cultures were softly shaken at 37°C for 2 h and the primary microglia-enriched supernatant was subcultured in the same medium for 2 days before the experiments. Cultures of microglia showed >95% of microglial marker Iba1-positive cells by immunocytochemistry.

BV-2 cells (AcceGen Biotechnology, Fairfield, NJ, United States) were cultured in 25 cm^2^ Nunc EasYFlask cell culture flasks (Thermo Fisher Scientific, Waltham, Massachusetts, United States) using RPMI-1640 culture medium (Sigma Aldrich, Missouri, United States) supplemented with 10% inactivated fetal bovine serum (Gibco, GranIsland, New York, United States), 2 mM glutamine, 100 U/mL penicillin and 100 μg/mL streptomycin (complete culture medium).

#### 2.8.2 EVs-cell interactions and cytokine expression analyses

For primary microglial cultures, 2.5 × 10^4^ cells were seeded in in 6-well plates (Thermo Fisher Scientific, Waltham, Massachusetts, United States), using DMEM with 4.5 g/L D-glucose, 4 mM glutamine, 10% fetal bovine serum, 10% horse serum and 100 U/mL penicillin, 100 μg/mL streptomycin (all reagents from GIBCO, GranIsland, New York, United States). After 72 h of incubation at 37°C, 50% of the culture medium in each well was removed and 25 μg of EVs of each *N. fowleri* isolate was suspended in fresh culture medium and added to the cells. In this case, the incubations were performed for 48 h, and, after this time, the supernatants were removed and the cells were lysed and homogenized using TRIzol reagent (Thermo Fischer Scientific, Waltham, Massachusetts, United States) for RNA purification, which was performed following the manufacturer’s instructions. Incubation of cells with bacterial 100 ng/mL lipopolysaccharide (LPS, serotype 0111: B4; Sigma Aldrich, Missouri, United States) and with the complete culture medium were also included as control of cell stimulation.

For BV-2 cell line, 2.5 × 10^5^ cells were seeded in 6-well plates (Thermo Fisher Scientific, Waltham, Massachusetts, United States) with RPMI-1640 culture medium (Sigma Aldrich, Missouri, United States) supplemented with 10% inactivated fetal bovine serum (Gibco, GranIsland, New York, United States), 2 mM glutamine and antibiotics (penicillin/streptomycin). After 24 h of incubation at 37°C, the culture medium was removed and cells were incubated with 25 μg of EVs of each *N. fowleri* isolate, suspended in complete culture medium and incubated for different time points: 4, 24 and 48 h. After each incubation time, the culture medium was removed and TRIzol reagent (Thermo Fischer Scientific, Waltham, Massachusetts, United States) was added for RNA extractions, which were performed as previously mentioned.

After RNA extractions, an incubation with DNase I, RNase-free (Thermo Fischer Scientific, Waltham, Massachusetts, United States) was performed according to the manufacturer’s recommendations and, once the RNAs were purified and quantified, expression analyses of genes *IL-1β, IL-6, IL-10, IL-12, IL-13, IL-18, IL-23, TNF-α, IFN-γ, TGF-β* and nitric oxide synthase (*NOS*) were performed by qRT-PCR (primer sequences are shown in Supplementary Table S1). For this purpose, the iTaq^™^ universal SYBR^®^ Green one-step universal SYBR^®^ kit (BioRad, Hercules, CA, United States) was employed, using *gapdh* and *actin* as reference genes. The sequences of the primers employed in this analysis are listed in Supplementary Table S1; primer sequences were located across exon–exon borders, avoiding any interspecifically and intraspecifically variable positions. Moreover, a calibration curve was performed according to Gómez-Samblas et al. (2018) to calculate the efficiency of each pair of primers.

Reactions were performed in a CFX-96 qRT-PCR system (BioRad, Hercules, CA, United States), using a final volume of 10 μL, which included 300 nM of each primer and 50 ng of RNA per reaction. The thermal cycling conditions consisted of retrotranscription at 50°C for 10 min, followed by an enzymatic activation step and DNA denaturation at 95°C for 1 min, 40 cycles of denaturation at 95°C for 10 s and annealing and extension steps at 60°C for 30 s, followed by plate reading. At the end of the qRT-PCR reactions, a melting gradient was applied from 65°C to 95°C in 0.5°C increments. Cytokine expression results were normalized against *gapdh* and *actin,* as well as the negative control (cells in complete culture medium).

#### 2.8.3 Analysis of morphological changes induced by EVs of *Naegleria fowleri* in culture primary culture from mouse cell microglia using immunofluorescence

Primary cultures of mouse brain microglia were prepared as previously described. Briefly, primary microglia (2.5 × 10^4^ cells) were seeded onto 12 mm-diameter round glass coverslips coated with 0.1 mg/mL poly-D-lysine and cultured in DMEM with 4.5 g/L D-Glucose, 4 mM glutamine, 10% fetal bovine serum, 10% horse serum and 100 U/mL penicillin, 100 μg/mL streptomycin for 2 days before the experiments. Cell stimulation was induced by 100 ng/mL LPS and 25 μg of EVs of each *N. fowleri* isolate suspended in the culture medium for 24 and 48 h.

To analyze microglial cell morphology, microglial cells were fixed with 4% cold paraformaldehyde in PBS for 20 min. After a washing step using PBS, the cells were permeabilized with 0.2% Triton X-100 in PBS for 10 min, blocked with 3% bovine serum albumin in PBS for 1 h, and stained with isolectin B4 from *Griffonia simplicifolia* (GS-IB4) Alexa Fluor 488 conjugate (Thermo Fisher Scientific, Waltham, Massachusetts, United States) diluted 1:100 in PBS for 1 h. DAPI staining was used to visualize nuclei. Coverslips were mounted in slides with FluorSave Reagent (Millipore, Burlington, Massachussetts, United States) and images were taken using a Zeiss Axiophot fluorescent microscope.

Images were analyzed by Image J software (version 1.50i, NIH). The cell morphology was studied by determination of the aspect ratio, as the ratio of width to height, of individual cells. Values of aspect ratio start at 1.0, which indicates a circle, while ascending values indicate enhanced cell ramification and elongation.

### 2.9 Determination of nucleic acids of *Naegleria fowleri* in EVs and PCR

In order to evaluate the presence of nucleic acids of *N. fowleri* in the EV fractions, samples of conditioned media (supernatants collected after the 5-h incubation period of trophozoites of the amoeba in 2% casein hydrolysate), and from the pellets obtained after the 16,000 × g (larger vesicles) and the 120,000 × g centrifugations (EV fraction, enriched in exosomes) from the EV isolation protocol were submitted to DNA extractions using phenol:chloroform:isoamyl alcohol (25:24:1) (Sigma Aldrich, Missouri, United States), following the manufacturer’s instructions. For these experiments, 50 mL of conditioned media collected for EV isolation (from approximately 7.9 × 10^7^ trophozoites) were employed and DNA samples were finally suspended in 40 μL nuclease-free water. DNA concentration was determined using a NanoDrop 2000 spectrophotometer (Thermo Fisher Scientific, Waltham, Massachusetts, United States).

After nucleic acid quantification of the samples, amplification of the ITS region of Vahlkampfiids was performed by PCR, using the pair of primers Vahl-F and Vahl-R, as previously described. Besides, *N. fowleri* species-specific conventional and quantitative PCR (qPCR) using primers NfITS-F and NfITS-R were also performed, according to the protocols described by Retana Moreira et al. (2020b) and including at least 12 DNA dilutions. Negative controls (template DNA replaced with distilled water) and positive controls (DNA extracted from trophozoites of *Naegleria fowleri*) were also included.

Amplification reactions of conventional PCRs were run in a Biometra TOne thermal cycler (Labgene Scientific, Châtel-Saint-Denis, Switzerland) and visualization of PCR products was performed using 1% agarose gels with SYBR safe DNA gel stain (Invitrogen, Waltham, Massachusetts, United States). For qPCR, the StepOne Real Time PCR System (Thermo Fischer Scientific, Waltham, Massachussetts, United States) was employed.

## 3 Results

### 3.1 Characterization of extracellular vesicles secreted by two clinic isolates of *Naegleria fowleri*

#### 3.1.1 Transmission electron microscopy, scanning electron microscopy and nanoparticle tracking analyses

The production of extracellular vesicles secreted by trophozoites of two clinic isolates of *Naegleria fowleri* was confirmed by TEM and NTA, after applying the isolation protocol described. Under our incubation conditions, amoebae produced approximately 0.94 μg/μL (*N. fowleri* Guanacaste) and 1.02 μg/μL (*N. fowleri* Limón) protein in extracellular vesicles and, after the 5 h incubation, viability of trophozoites was not affected.

**Figure 1:**
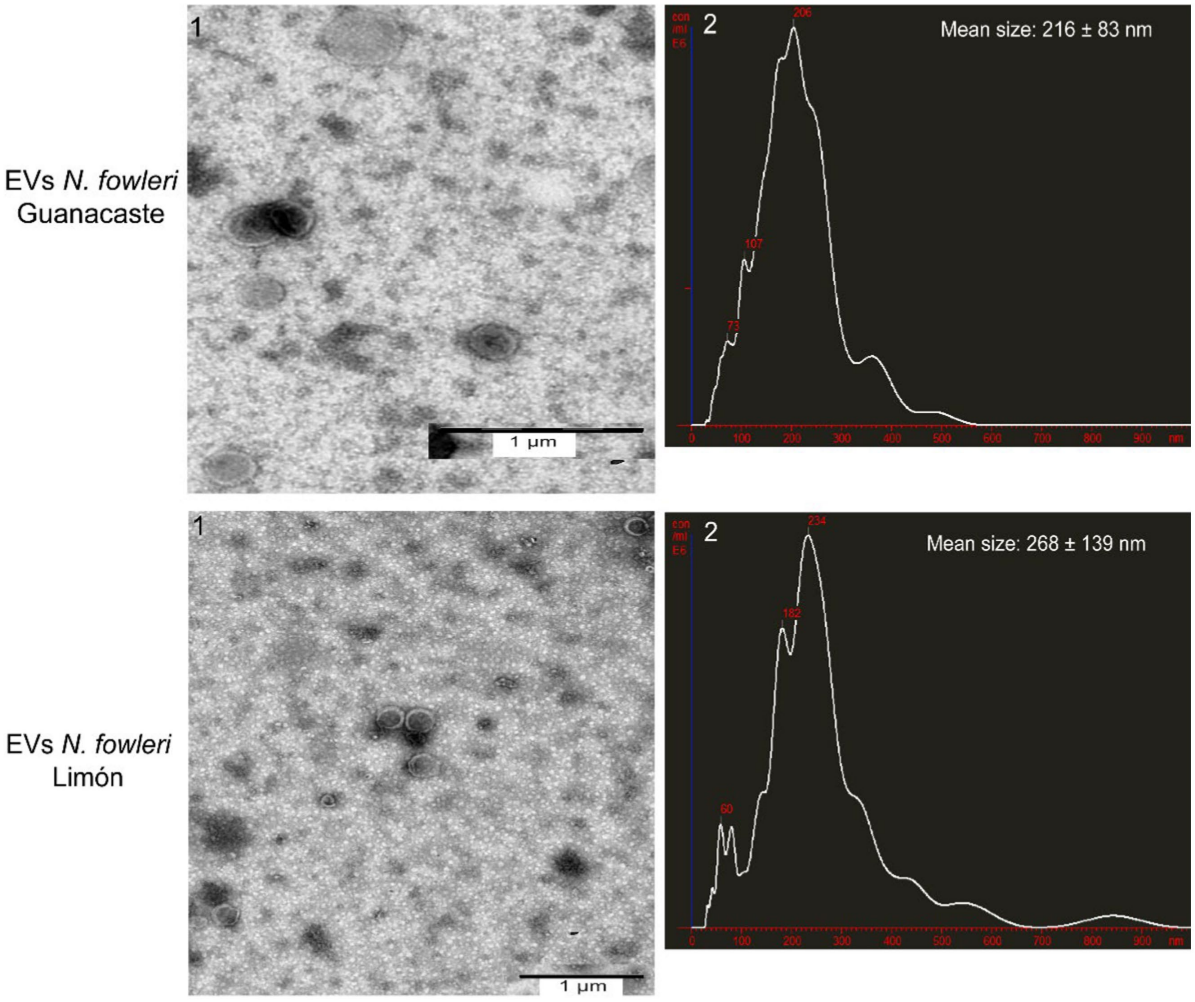
Transmission electron microscopy images and nanoparticle tracking analysis graphs of extracellular vesicles secreted by trophozoites of two clinic isolates of *Naegleria fowleri*. Typical cup shaped EVs of different diameters are produced by both isolates; hydrodynamic mean sizes of EVs obtained were 216 ± 83 nm in *N. fowleri* Guanacaste and 268 ± 139 nm in *N. fowleri* Limón.

Figure 1 shows representative transmission electron microscopy images and nanoparticle tracking analysis of extracellular vesicles secreted by both isolates, after applying the isolation procedure. NTA revealed a mean hydrodynamic size of extracellular vesicles of 216 nm ± 83 nm and a mode of 206 nm for *N. fowleri*, as previously described (Retana Moreira et al., 2022), while the mean size of EVs secreted by *N. foweri* Limón was 268 ± 139 nm, with a mode of 234 nm. Using the same methodology, it was also possible to determine that 5 × 10^7^ trophozoites of *N. fowleri* Guanacaste secreted 4.96 × 10^8^ particles/mL, while trophozoites of *N. fowleri* Limón secreted 3.2 × 10^8^ particles/mL.

**Figure 2:**
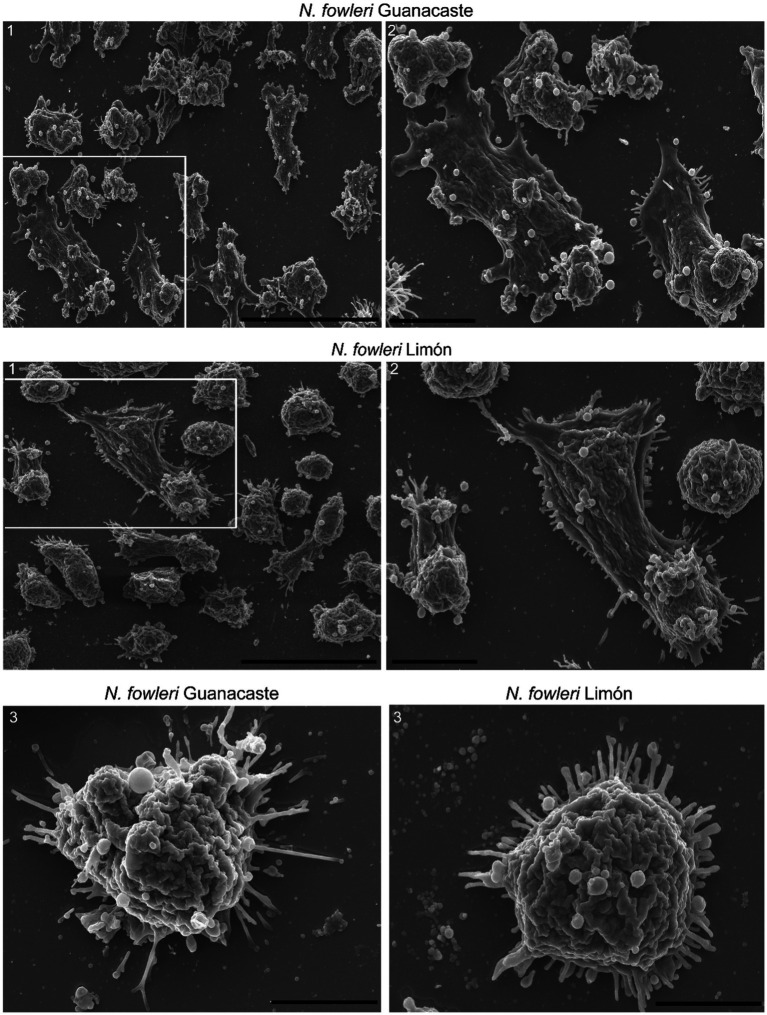
Scanning electron microscopy of trophozoites of *Naegleria fowleri* Guanacaste and *N. fowleri* Limón that confirms the secretion of extracellular vesicles. In these images, individual and grouped EVs of different sizes can be observed surrounding the trophozoites, as well as vesicles of variable sizes being released from different regions of the plasma membrane. In these Figure correspond to magnifications of 1. Scale bars: 40 μm (1), 10 μm (2), and 5 μm (3).

Scanning electron microscopy also confirmed the secretion of extracellular vesicles by both clinic isolates of *Naegleria fowleri*, as shown in Figure 2. In these images, it is possible to observe individual vesicles and clusters of different sizes surrounding the trophozoites. Vesicles of variable sizes being released from different regions of the plasma membrane are also observed (Figure 2).

### 3.2 Protein pattern and recognition of extracellular vesicles by polyclonal anti-*Naegleria fowleri* antibodies

**Figure 3:**
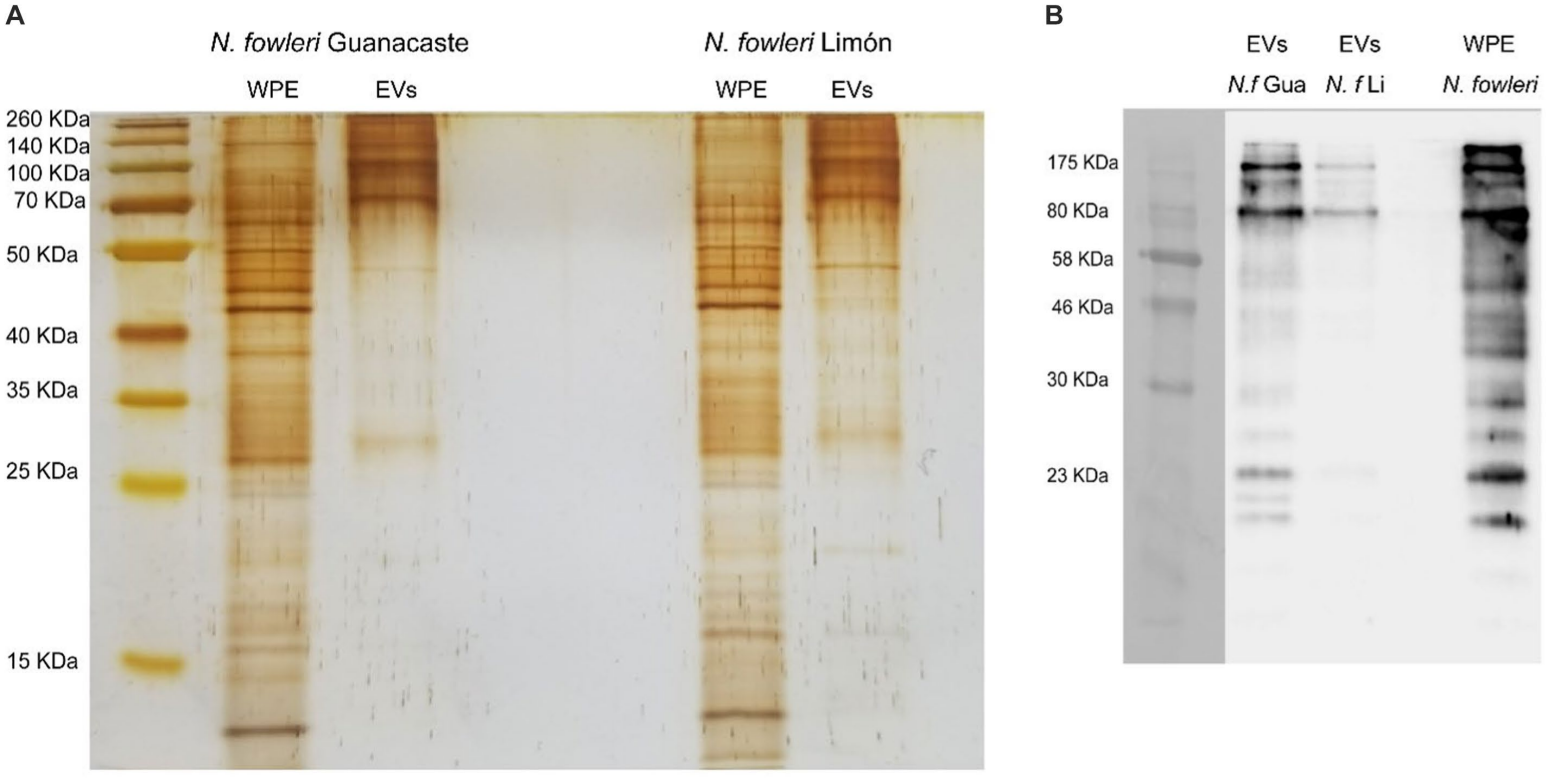
Protein profile of extracellular vesicles secreted by *Naegleria fowleri* and recognition by polyclonal anti-*Naegleria fowleri* antibodies: **(A)** Silver staining apparently showing similar band patterns in extracellular vesicles from isolates Guanacaste and Limón, which range from >15 kDa to 260 kDa. **(B)** Western blot that shows the recognition of different bands in EVs of each isolate. In this sense, bands over 70–80 kDa were highly recognized in EVs of both isolates by the polyclonal antibodies; however, recognition of more protein bands was observed in EVs secreted *N. fowleri* Guanacaste (Retana Moreira et al., 2022). For silver staining, approximately 9 μg of protein/EV sample were loaded onto the gel; for Western blot, approximately 6 μg of protein/EV sample were loaded onto the gel. WPE, whole protein extracts of trophozoites of *N. fowleri*; EVs, extracellular vesicles.

The protein profile of extracellular vesicles secreted by trophozoites of both isolates of *Naegleria fowleri* after silver staining is shown in Figure 3 and Supplementary Figure S1, in which protein bands ranging from approximately >15 kDa to 260 kDa were identified. In this Figure, high molecular weight bands seem to be more predominant in EVs from both isolates.

Western blot analysis using polyclonal anti-*Naegleria fowleri* antibodies confirmed the recognition of proteins of *N. fowleri* in lysates of trophozoites employed to produce the antibodies (Supplementary Figure S2), as well as the recognition of *N. fowleri* proteins in extracellular vesicles secreted by both isolates. In this sense, recognition of EV bands was observed from over 10 kDa to 175 kDa, as previously reported.

### 3.3 Cytokine expression analyses

**Figure 4:**
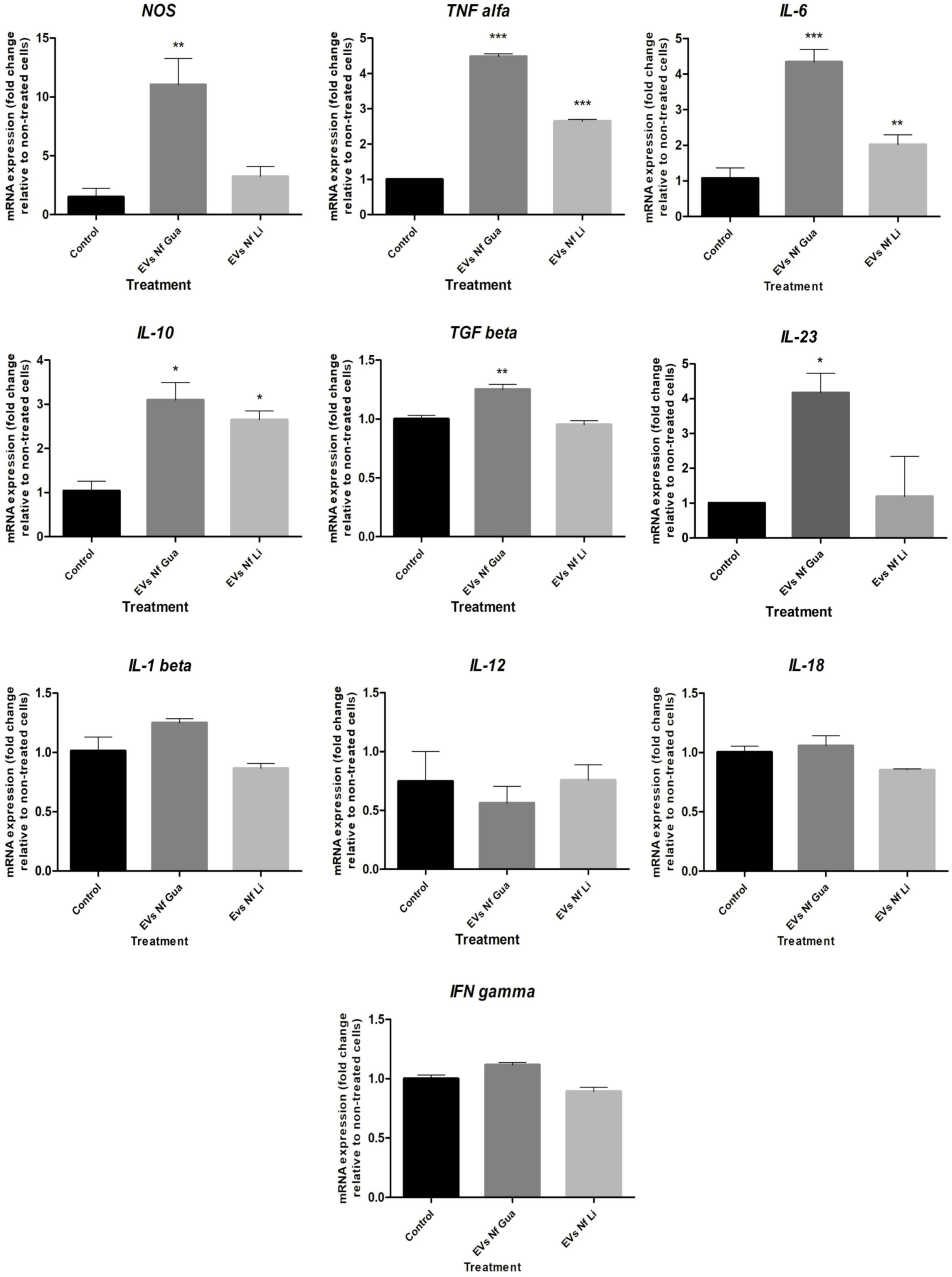
Differential mRNA expression analyses of *NOS* and cytokines after the incubation of primary culture of mouse brain microglia with extracellular vesicles secreted *by Naegleria fowleri* Guanacaste and *N. fowleri* Limón. Primary cultures of mouse brain microglia were stimulated with extracellular vesicles (25 μg) of two clinic isolates of *N. fowleri* for 48 h and qRT-PCRs were performed to analyze the expression pattern of *NOS* and cytokines. Values are presented as the mean ± SD and one-way ANOVA with Tukey *post hoc* test was performed for multiple comparisons to the negative control without treatment. ***p < 0.0005, **p < 0.005, *p < 0.05.

*NOS* and cytokine expression analyses performed after the incubation of primary cultures of mouse brain microglia with EVs of each isolate of *N. fowleri* for 48 h are shown in Figure 4. In this Figure, an upregulation of *NOS* and proinflammatoy cytokines *IL-6, IL-23*, and *TNF-α* was observed, as well as the increased expression of *IL-10*, the latter a regulatory cytokine. In all cases, transcription levels of genes were significantly higher than the expression levels found in control cells, especially when the isolate *N. fowleri* Guanacaste was employed. LPS activation of cells and its cytokine expression analysis is shown in Supplementary Figure S3.

**Figure 5:**
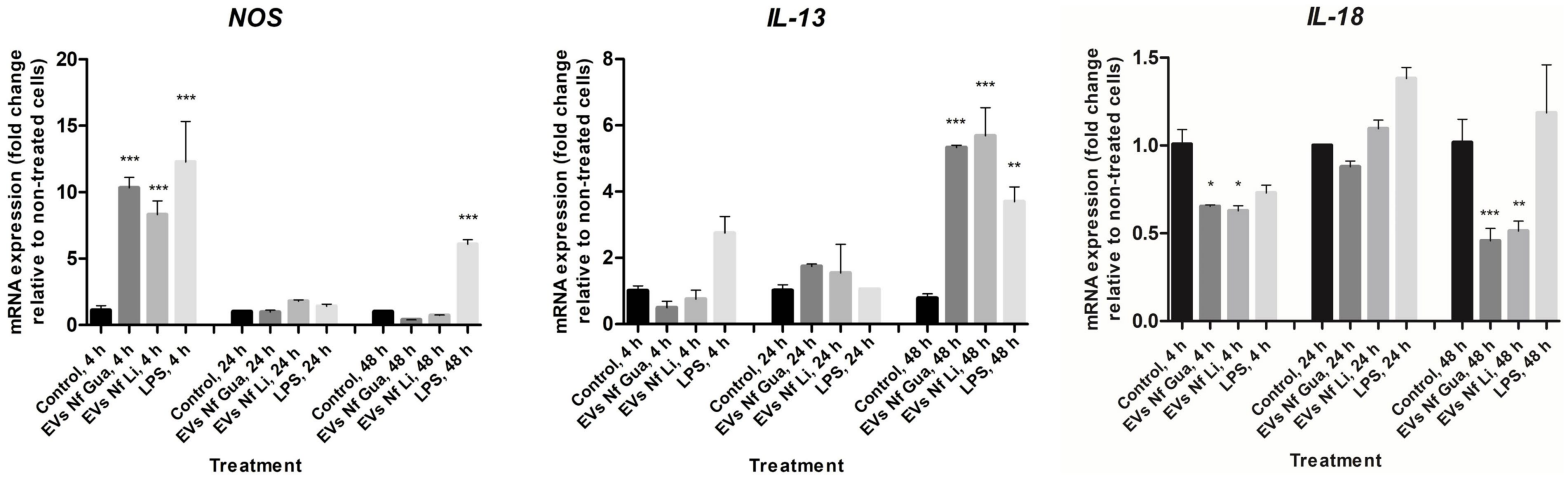
Differential mRNA expression analyses of *NOS*, *IL-13*, and *IL-18* after the incubation of BV-2 cells with extracellular vesicles secreted *by Naegleria fowleri* Guanacaste and *N. fowleri* Limón. BV-2 microglial cells were stimulated with extracellular vesicles (25 μg) of two clinic isolates of *N. fowleri* for 4, 24, and 48 h and qRT-PCRs were performed to analyze the expression pattern of *NOS* and cytokines. Values are presented as the mean ± SD and one-way ANOVA with Tukey *post hoc* test was performed for multiple comparisons to the negative control without treatment. ***p < 0.0005, **p < 0.005, *p < 0.05.

For BV-2 cells, results of interleukin expression after the incubation with EVs of the two isolates of *N. fowleri* revealed a downregulation in the expression of *IL-18* and an upregulation for *IL-13* 48 h after the stimuli (Figure 5). However, non-statistically significant differences were found in the expression levels of the rest of cytokines assayed with respect to control cells (Supplementary Figure S4). For *NOS*, transcription levels were significantly higher after 4 h of incubation of cells with the EVs; moreover, these differences were not found after 24 and 48 h of incubation.

### 3.4 Microglial cell morphology analysis using fluorescence microscopy

**Figure 6:**
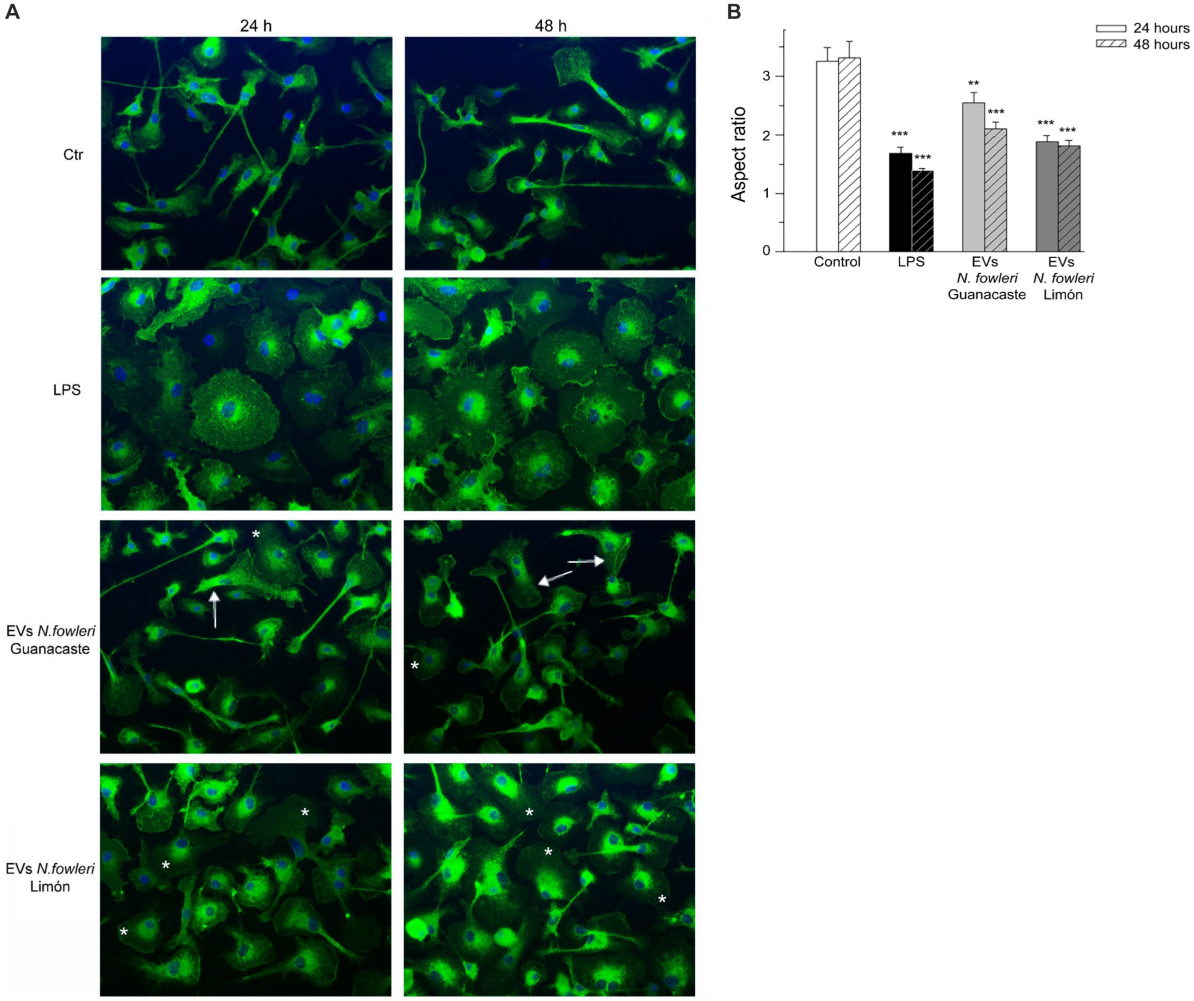
Morphological changes of primary microglia from mouse brain stimulated with LPS or with extracellular vesicles secreted by two isolates of *Naegleria fowleri*. **(A)** GS-IB_4_ Alexa Fluor 488 conjugate (green) was used to visualize microglial cell morphology and DAPI staining (blue) to visualize nuclei. Cells showed the expected change in morphology from control microglia in culture with small cellular bodies and long processes (Ctr) to LPS-stimulated microglia with amoeboid morphology (LPS). While cells incubated with EVs isolated from *N. fowleri* Guanacaste barely showed a morphological change except for lamellipodia expansion (arrows), cells incubated with EVs from *N. fowleri* Limón strongly reacted and showed processes retraction and a more amoeboid morphology (*). Moreover, similar results were found after 24 and 48 h of incubation. Scale bar: 30 μm. **(B)** Determination of the aspect ratio parameter in control cells, LPS-activated microglia, and cells stimulated with EVs from the two *N. fowleri* isolates. Values of aspect ratio start at 1.0, which indicates a circle, while ascending values indicate enhanced cell ramification and elongation. Data are presented as the mean ± SEM and one-way ANOVA with Tukey *post hoc* test was performed for multiple comparisons to the negative control for each time point. ***p < 0.0005, **p < 0.005.

In order to perform an analysis of inflammatory phenotype of microglia, primary microglia from newborn mouse brains were incubated with EVs of the two isolates of *N. fowleri;* the bacterial endotoxin LPS-induction in primary microglia was also included (Figure 6). GS-IB4 staining showed the characteristic change in morphology from homeostatic microglia, with small cellular body and long processes in control cultures, to LPS-stimulated microglia with a more amoeboid morphology (Figure 6A), as it was determined in terms of the aspect ratio parameter (Figure 6B) However, evident changes in the morphology of cells were observed after the incubation with EVs of each isolate of *N. fowleri*. In this sense, cells incubated with EVs from *N. fowleri* Limón exhibited a clear morphological change to a more rounded shape, more similar to that found under LPS conditions. However, the incubation with EVs isolated from *N. fowleri* Guanacaste induced changes in morphology to a lesser extent, since cells still showed long processes, but with larger lamellipodia than controls. Similar results were found between 24 and 48 h of cell incubation with the stimuli.

### 3.5 Detection of DNA of *Nagleria fowleri* in EV fractions

**Figure 7:**
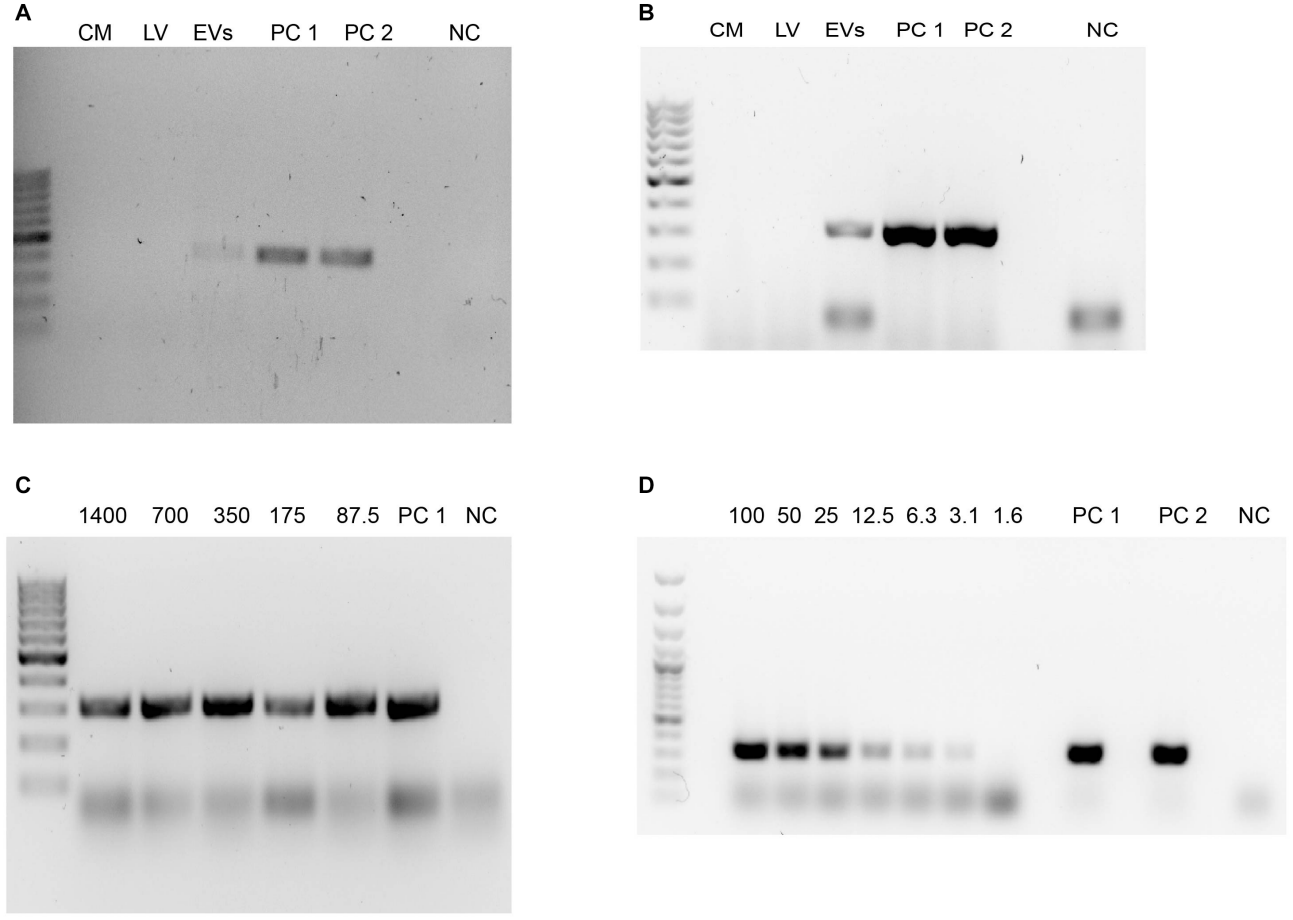
Detection of DNA from *Naegleria fowleri* in the EV fractions using phenol:chloroform:isoamyl alcohol extractions and conventional PCR. **(A)** Agarose gel electrophoresis after the amplification of the ITS region of Vahlkampfiids using Vahl-F and Vahl-R primers. **(B)** Agarose gel electrophoresis after the amplification of the ITS region of *N. fowleri* using NfITS-F and NfITS-R primers. For these PCRs, the same amount of DNA (700 ng) was employed in each sample. **(C,D)** Agarose gel electrophoresis after the amplification of the ITS region of *N. fowleri* using NfITS-F and NfITS-R primers, in which serial dilutions of the DNA obtained from the EV fraction were employed (expressed in ng). CM, conditioned media; LV, 16,000 × g pellet sample that contains larger vesicles; EVs: pellet of extracellular vesicles obtained after the 120,000 × g ultracentrifugation; PC 1: DNA from trophozoites of *N. fowleri* Guanacaste (positive control); PC 2: DNA from trophozoites of *N. fowleri* Limón (positive control); NC: negative control. GeneRuler 100 bp Plus DNA ladder (Thermo Fischer Scientific, Waltham, Massachussetts, United States) was employed as the DNA ladder.

Figure 7 reveals conventional PCR results in which DNA from the EV fractions were employed. In this sense, conditioned media and samples of pellets obtained after the 16,000 × g and 120,000 × g centrifugation steps for EV isolation were submitted to phenol:chloroform:isoamyl alcohol DNA extractions, for further analyses of *N. fowleri* specific DNA amplification using family (Vahlkampfiid) and *N. fowleri* species-specific primers. In Figures 7A,B, conditioned media (lane 1) and the pellets obtained after the 16,000 × g centrifugation (lane 2) resulted negative for Valhkampfiids and *N. fowleri* at the DNA amounts employed (700 ng) while the pellets corresponding to DNA obtained from the EV fraction resulted positive, demonstrating the presence of bioactive *N. fowleri*-specific DNA in these fractions.

To confirm this result, another species-specific PCR using primers NfITS and applying serial dilutions to the DNA extracted from the EV fraction was performed, resulting in amplification until employing a DNA concentration of 3.125 ng, the last dilution in which the specific conventional PCR for *N. fowleri* resulted positive (Figures 7C,D). These results were confirmed with a *N. fowleri* species-specific qPCR (Retana Moreira et al., 2020a,b), in which mean CT = 30 for the dilution that contained 0.4 ng of DNA, mean CT = 31.5 for the dilution that contained 0.2 ng of DNA and mean CT = 32 for the dilution that contained 0.1 ng DNA.

## 4 Discussion

Extracellular vesicles are a heterogeneous group of vesicles delimited by a lipid bilayer and are considered part of the excretion/secretion products. These vesicles are released by almost all cell types and have a pivotal role in intercellular communication, as they have been considered “delivery trucks” that transport diverse molecules from one cell to another, inducing changes at different levels in the recipient cell (Dong et al., 2021). A role of EVs in the pathogenesis of several diseases has also been proposed by different authors, which supports their study and evaluation as potential biomarkers. In this sense, it has been demonstrated the presence of virulence factors as part of the cargo of the vesicles, related to adhesion, invasion and survival processes, as well as the modulation of the immune response they could exert (Nievas et al., 2020).

Isolated from most organisms, the study and characterization of EVs in protozoan parasites has gained considerable interest, mostly in the case of infections that lack efficient diagnostic tools and effective treatments (Dong et al., 2021; Cruz Camacho et al., 2023); primary amoebic meningoencephalitis, caused by the free-living amoeba *Naegleria fowleri*, is one of this type of infections. Primary acute meningoencephalitis is a necrotizing haemorragic meningoencephalitis of acute and fulminant course, with a letality rate of 97–98% (Siddiqui et al., 2016). In this infection, the trophozoite stage of the amoeba enters the host through the nasal passage and reaches brain tissue throughout the olfactory bulb as early as 24 h post inoculation (Jarolim et al., 2000). The establishment of the amoeba in the brain induces an intense inflammatory response that, along with the mechanic damage directly provoked by the microorganism (Cervantes-Sandoval et al., 2008; Lertjuthaporn et al., 2022), are responsible of the extensive lesions observed. Previous studies have demonstrated that excretion/secretion products, including EVs, are involved in the pathogenic processes of infections, mainly by the stimulation and modulation these products could exert over the immune response of the host (Kim et al., 2009). In this work, a characterization of the extracellular vesicles secreted by two clinic isolates of *N. fowleri* was performed, and the effect these vesicles exert over microglial cells was analyzed.

For the purpose of our analyses, EVs from trophozoites of two clinic isolates of *N. fowleri* were obtained after applying differential centrifugation, filtration and ultracentrifugation of conditioned media obtained after 5 h of incubation. Size ranges of the vesicles obtained, analyzed by NTA, revealed particles of 216 ± 83 nm (EVs of *N. fowleri* Guanacaste) and 268 ± 139 nm (EVs of *N. fowleri* Limón). These sizes coincide with previous studies performed by our research group (Retana Moreira et al., 2022), but result slightly higher to the sizes reported by Lertjuthaporn et al. (2022). Moreover, scanning electron microscopy images show the secretion of individual EVs, as well as EVs forming small clusters, that appear to be released through the plasma membrane of trophozoites of amoebae. With the methodologies employed for characterization of EVs in this study, it was not possible to observe statistically significant differences between the vesicles secreted by both clinical isolates.

To evaluate the presence of proteins in EVs that could be recognized by the antibodies obtained after the immunization, SDS-PAGE electrophoresis and Western blot were employed, the latter using polyclonal anti-*N. fowleri* antibodies produced in rats against a complete extract of trophozoites *of N. fowleri* (ATCC *N. fowleri* Carter 30808). Results obtained after these analyses also revealed similarities in both isolates (Figure 3), in which antibody recognition was confirmed. In these experiments, a lysate of trophozoites of *N. fowleri* was also included and the recognition of bands by the antibodies was also observed, a result that evidences that EVs of both isolates of *N. fowleri* include cytosolic and plasma membrane components of the trophozoites that are recognized by the antibodies obtained. More studies are necessary to identify potential significant differences between the cargo of EVs secreted by both isolates.

Regarding band components above 80 kDa, the recognition of 3 bands was clearly observed. Previous proteomic analyses performed by our research group (Retana Moreira et al., 2022) demonstrated the presence of components of the plasma membrane and cytoskeleton in these bands, as well as some virulence factors reported in infections with this and other species of parasites, that can eventually activate or modulate the immune response, such as leucine aminopeptidase and elongation factor 1-alpha (eeEF1-α), the latter an important factor for immunosuppression and priming of host cells for *Leishmania* invasion (Silverman and Reiner, 2011; Vyas et al., 2014; Timm et al., 2017).

Since brain tissue is the target during a *N. fowleri* infection, we decided to evaluate the possible role of EVs of *N. fowleri* in the immune modulation of microglial cells. These are highly specialized resident cells responsible of monitoring brain microenvironment, detecting and responding to any type of tissue damage, infections, or homeostatic disturbance (Nimmerjahn et al., 2005). Microglia stimulation may induce a drastic change in cellular morphology and, to evidence possible cell responses after the stimulus with EVs *N. fowleri*, we employed isolectin-B4 fluorescence staining for the observation of morphological changes in microglia. In this sense, we employed primary microglia obtained from newborn mice brain in order to have a more physiological approach compared to transformed cell lines. Results revealed a change from a ramified cell with small cellular bodies and long processes (related to a homeostatic microglial state), to an amoeboid morphology more characteristic of a pro-inflammatory phenotype, including transient states that reflect functional events related to the disturbing fact (Colonna and Butovsky, 2017). In this study, when primary microglia was challenged with EVs secreted by *N. fowleri,* evident morphological changes were observed when compared to control cells, especially when EVs from *N. fowleri* Limón were employed, which confirmed the proinflammatory microglial response induced by EVs produced by amoebae.

Cell responses to EVs secreted by *N. fowleri* were also quantitatively assayed, determining changes in the expression levels of cytokines and *NOS* using qRT-PCR, which revealed increased levels of *NOS* and proinflammatory cytokines *IL-6*, *IL-23*, and *TNF-α*, as well as increased levels of the regulatory cytokine *IL-10.* In all cases, statistically significant differences were observed when compared to control unstimulated cells, especially when EVs of the isolate Guanacaste were employed. Similar results -but registered during the first 12 h after the stimulus- were reported by other authors when rat primary microglia cells were co-cultured with trophozoites of the amoeba (Oh et al., 2005), or when excretory/secretory products were employed (Lee et al., 2011), reinforcing the idea that these cells (and the cytokines they produce after the contact with trophozoites of *N. fowleri* or its excretory/secretory products) have a crucial role in the exacerbated inflammatory response observed during PAM. Moreover, the concomitant and very marked increase in the expression of *TNF-a, NOS*, and *IL-6* in the primary microglial culture suggests a *N. fowleri* EV-induced polarization of the cells to a proinflammatory profile, capable of secreting destructive factors that contribute to neuronal damage. A more discrete augmentation in the regulatory cytokines *IL-10* and *TGF-β* was also observed, and these are markers of more anti-inflammatory phenotype, the other side of the spectrum. Although it may appear as a contradiction, the plasticity of the functional polarization in microglial cells could provide some clarity. In contrast to the behavior in non-CNS sites, where macrophages tend to first acquire proinflammatory characteristics upon the harmful stimulus and then, the response evolves toward a regulatory profile, in the CNS this process is inversed (Hu et al., 2015). Moreover, and according to Saraiva and O'Garra (2010), IL-10 could be induced in many situations in which proinflammatory cytokines are also induced, despite the pathways that induce its expression can regulate the expression of those cytokines in time. Park et al. (2007) demonstrated the endogenous production of IL-10 by rat microglial cells after the stimulation with LPS; interestingly, IL-10 was simultaneously produced with proinflammatory cytokines like IL-1*β,* TNF- *a* and iNOS. The authors propose that this endogenous production of IL-10 could help to control the production of inflammatory mediators in microglia in an autocrine way, resulting in neuroprotection even in the early stage of an acute inflammation of the brain. Due to the technical challenges of primary microglial culture and to minimize the number of animals used in the experiments, a kinetic evaluation of the markers’ expression was not performed, nor the use of different doses of EVs was included in this study, but it would be suggested to evaluate this aspect. A further consequence of this expression profile could be the priming of CD4+ T lymphocytes for a Th17 adaptive response, a response that has not been studied during a *N. fowleri* infection, but it has been shown to amplify the inflammatory response in murine models of CNS inflammation (Rostami and Ciric, 2013). As stated by Moseman (2020), there are many open questions surrounding fundamental immunological processes during a *N. fowleri* infection, so that more studies should be carried out to clarify the contribution of the early proinflammatory response by microglial cells to the immune-mediated pathological mechanisms of PAM.

Antiparasitic effects of nitric oxide in infections caused by helminths and protozoan parasites are widely known and described; however, it has also been postulated its role in producing damage during this type of infections (Brunet, 2001; Omar and Abdelal, 2022). Regarding *N. fowleri,* the first published results related to this topic revealed that activated macrophages could destroy the amoebae by an arginase-dependent cytolytic mechanism that results in NO production (Fischer-Stenger and Marclano-Cabral, 1992). However, using an *in vitro* model, Rojas-Hernández et al. (2007) demonstrated that this amoeba was highly resistant to NO-destruction, suggesting that its production during PAM could contribute to tissue damage instead of amoeba destruction. Regarding TNF-α, its transcendental role in increasing the oxidative burst in neutrophils, as well as the release of lysosomal enzymes in response to the amoeba has also been reported (Ferrante et al., 1988; Michelson et al., 1990). Moreover, tentative anti-amoebic roles of IL-6 are still unclear, even suggesting that its upregulation during the infection could contribute, as a secondary effect, to the tissue damage observed in the brain during PAM (Chen and Moseman, 2023).

In this work, if we try to compare transcription levels of mRNA of cytokines evaluated after the incubation of primary cultures from mouse cell microglia and BV-2 cells with EVs of *N. fowleri* for 48 h, both similarities and differences could be found. For example, while expression levels of *IL-12* and *IFN-γ* were not statistically different when compared to the controls for both cell types, an upregulation of *NOS*, *IL-6*, and *IL-10* was found only in primary cultures of microglia; additionally, a downregulation of *IL-18* was observed only in BV-2 cells. These results, especially the differences observed, are expected when different cell types are employed, particularly if responses of primary cells are compared to responses of immortalized cell lines. While the use of a primary cell model could more accurately reflect what occurs in an infection *in vivo* with this amoeba, it is clear that the use of cell lines such as BV-2 is advantageous for the experimental work at a laboratory level, since its “immortality” allows its indefinite cultivation, facilitating the analysis and characterization of biological processes. In a recent publication, Lê et al. (2023) analyzed the immune response in BV-2 microglial cells upon incubation with EVs of *N. fowleri* (Carter NF69 strain, ATCC 30215) for 3, 6, and 9 h. In this work, besides *IL-6* and *TNF-α,* increased expression levels of *IL-1α, IL-1β, IFN-γ, MIP-1*, and *MIP-2* (but not IL-10) was also reported. The authors suggested that EVs of *N. fowleri* are pathogenic factors involved in contact independent pathogenic mechanisms of *N. fowleri* by inducing proinflammatory immune responses. In our study, and using the same cell line, increased expression levels of *NOS* (after 4 h of incubation with EVs of *N. fowleri*) and *IL-13* (after 48 h of incubation with EVs of *N. fowleri*) were observed, but not increased expression of *IL-1β* or *IFN-γ*, differences that could be explained due to differences in the incubation times employed and the individual characteristics of the *N. fowleri* clinical wild isolates in comparison to a reference strain.

It is also interesting to highlight the upregulation of *IL-13* and the simultaneous downregulation of *IL-18* expression found in our study using the BV-2 cell line. In a murine model of infection with the nematode *Nippostrongylus brasiliensis*, IL-13 was shown to inhibit the expression of IL-18, an inflammasome-activated cytokine that contributes to inflammation a cell death via pyroptosis (Chenery et al., 2021). Furthermore, IL-13 has been described as a key modulator of brain inflammation as it has been shown to induce microglial cell death to downregulate the inflammatory process and hinder neuronal damage and its blockade can increase the expression of TNF-α and iNOS (Shin et al., 2004), which is consistent with our findings on the kinetics of *NOS* and *IL-13* expression. This study suggests that IL-13 expression may act as a mechanism for neuronal survival; however, in the case of *N. fowleri*, it may be insufficient to compensate for the damaged induced by the initial proinflammatory response. The authors also described regulation of IL-13 secretion by other cells present in the brain’s microenvironment, such as neurons, so a next step in the study of the IL-13/iNOS and IL-13/IL-18 regulation should be the study in co-culture or animal models. Altogether, the results derived from our work confirm the immunostimulatory role of EVs secreted by *N. fowleri* over microglial cells, suggesting that the strong proinflammatory response observed during primary acute meningoencephalitis could have a fundamental role in the tissue damage observed in *in vivo* infections in less than 96 h after the infection with the amoeba (Rojas-Hernández et al., 2004).

Finally, the existence of DNA in EVs is now considered a consensus in the field of EVs (Ghanam et al., 2022). In all previous works regarding the characterization of EVs secreted by *N. fowleri* (Lertjuthaporn et al., 2022; Retana Moreira et al., 2022; Lê et al., 2023; Russell et al., 2023), the presence of bioactive nucleic acids as part of the EV cargo or in the EV fractions has not been analyzed. However, in this work, we report the preliminary finding of DNA in the EV fraction that corresponds to small EVs, detectable using a phenol:chloroform extraction and *N. fowleri* species-specific PCR protocols even in a concentration of 3.125 ng for conventional PCR. DNA-containing EVs from human cells have been well studied in terms of DNA loading in vesicles and their role in homeostasis, immunomodulation and gene transference (Chang et al., 2020; Malkin and Bratman, 2020; Elzanowska et al., 2021). Moreover, the presence of DNA in EVs has also been described in EVs secreted by other protozoan organisms (Tatischeff et al., 1998; Douanne et al., 2022). In this sense, and as this is a preliminary report, there are pending assays to ascertain the location of this DNA in the vesicles, the profile of the DNA content and its implication in host immune response and messaging with host-cells and other amoebae. Moreover, our group is currently working in applying different enzymatic treatments to the EV fractions to confirm that bioactive DNA in included within the smaller EVs, especially if it is taken into account that, at least in these preliminary experiments, bioactive *N. fowleri* DNA was not found in the larger vesicle fractions that are supposed to contain ectosomes, a type of EVs with a different cargo and formed through outward budding of the plasma membrane.

Regarding the DNA cargo in EVs secreted by trophozoites of *N. fowleri*, we suggest two prospective research opportunities: the DNA interaction with host cell machinery and its potential use as a biomarker in a clinical context. As described elsewhere, it has been shown that DNA-harboring EVs from malaria parasites could stimulate cytosolic pathogen DNA sensors in host monocytes to elicit a cytokine response (Sisquella et al., 2017). On the other hand, cancer related studies of EV-DNA in plasma and other body fluids for new liquid biopsy applications are a reality (Malkin and Bratman, 2020), and this sets a base for understanding and proposing that DNA from pathogens, included in EV fractions, could also be considered a molecular biomarker as it has been proposed in a study with chronic Chagas disease patients, in which the detection of *Trypanosoma cruzi* nuclear and kinetoplast DNA was performed in serum circulating EVs (Lozano et al., 2023). For the specific case of primary acute meningoencephalitis, a biomarker present in blood instead of cerebrospinal fluid could make the difference in timely and non-invasive diagnosis, and we suggest this could be studied as it has been found that DNA-carrying EVs could cross the intact blood–brain-barrier, being detectable in peripheral blood (García-Romero et al., 2017). Small RNAs have also been found in protozoan derived EVs (Sabatke et al., 2021), which also suggests that EV nucleic acids represent open novel approaches for parasitic diseases research.

## Data availability statement

The raw data supporting the conclusions of this article will be made available by the authors, without undue reservation.

## Ethics statement

The animal study was approved by the Ethics Committee of the University of Granada (Ethics Committee, 235- CEEA-OH-2018), as well as by the authorities of the Regional Government of the Junta de Andalucía with number 12/11/2017/162. The study was conducted in accordance with the local legislation and institutional requirements.

## Author contributions

LR: Conceptualization, Formal analysis, Funding acquisition, Investigation, Methodology, Supervision, Visualization, Writing – original draft, Writing – review & editing. AC-G: Conceptualization, Investigation, Methodology, Visualization, Writing – original draft, Writing – review & editing. MS: Conceptualization, Formal analysis, Investigation, Methodology, Writing – original draft, Writing – review & editing. SM-C: Formal analysis, Investigation, Visualization, Writing – original draft, Writing – review & editing. JA-O: Formal analysis, Investigation, Writing – original draft, Writing – review & editing. FC: Methodology, Writing – original draft. MJ: Methodology, Writing – original draft. AO: Conceptualization, Formal analysis, Funding acquisition, Investigation, Supervision, Writing – original draft, Writing – review & editing. EA: Conceptualization, Funding acquisition, Investigation, Methodology, Supervision, Visualization, Writing – original draft, Writing – review & editing.
