# Supplementary material for: Providing an in vitro depiction of microglial cells challenged with immunostimulatory extracellular vesicles of Naegleria fowleri
Source: Front Microbiol. 2024 Feb 5;15:1346021. doi: 10.3389/fmicb.2024.1346021 (PMC10876093; doi:10.3389/fmicb.2024.1346021)
Supplement: Supplementary file 1 [file Data_Sheet_1.pdf]

Supplementary information

Table S1. Sequences of the forward and reverse primers employed for gene amplification and expression analyses

Gene	Forward sequence (5'-3')	Reverse sequence (5'-3')
IL-1β	TGCCACCTTTTGACAGTGATG	CTCTTGTTGATGTGCTGCTG
IL-6	AGTTGCCTTCTTGGGACTGA	TCCACGATTTCCCAGAGAAC
IL-10	CAGAGCCACATGCTCCTAGA	TCATTTCCGATAAGGCTTGG
IL-12	GACCAAACCAGCACATTGAA	CTACCAAGGCACAGGGTCAT
IL-13	CTGAGCAACATCACACAAGACC	AGGCTCCCCTTTGAAGATGT
IL-18	GACCAAACCAGCACATTGAA	CTACCAAGGCACAGGGTCAT
IL-23	TAATGTGCCCCGTATCCAGT	AGGCTCCCCTTTGAAGATGT
TNF-α	CCCCAAAGGGATGAGAAGTT	CACTTGGTGGTTTGCTACGA
IFN-γ	CACCCTGAAGTCGTTGTGAA	GATCTCCCCACTCCGGTTAT
TGFβ	TGGAGCAACATGTGGAACTC	AGCCTTGTATCCCGTCTCTT
NOS	TGACACACAGCGCTACAACA	CCATGATGGTCACATTCTGC
gapdh	ATGTGTCCGTCGTGGATC	ACCTGGTCCTCAGTGTAGC
actin	TCCATCATGAAGTGTGACGT	GAGCAATGATCTTGATCTTCAT

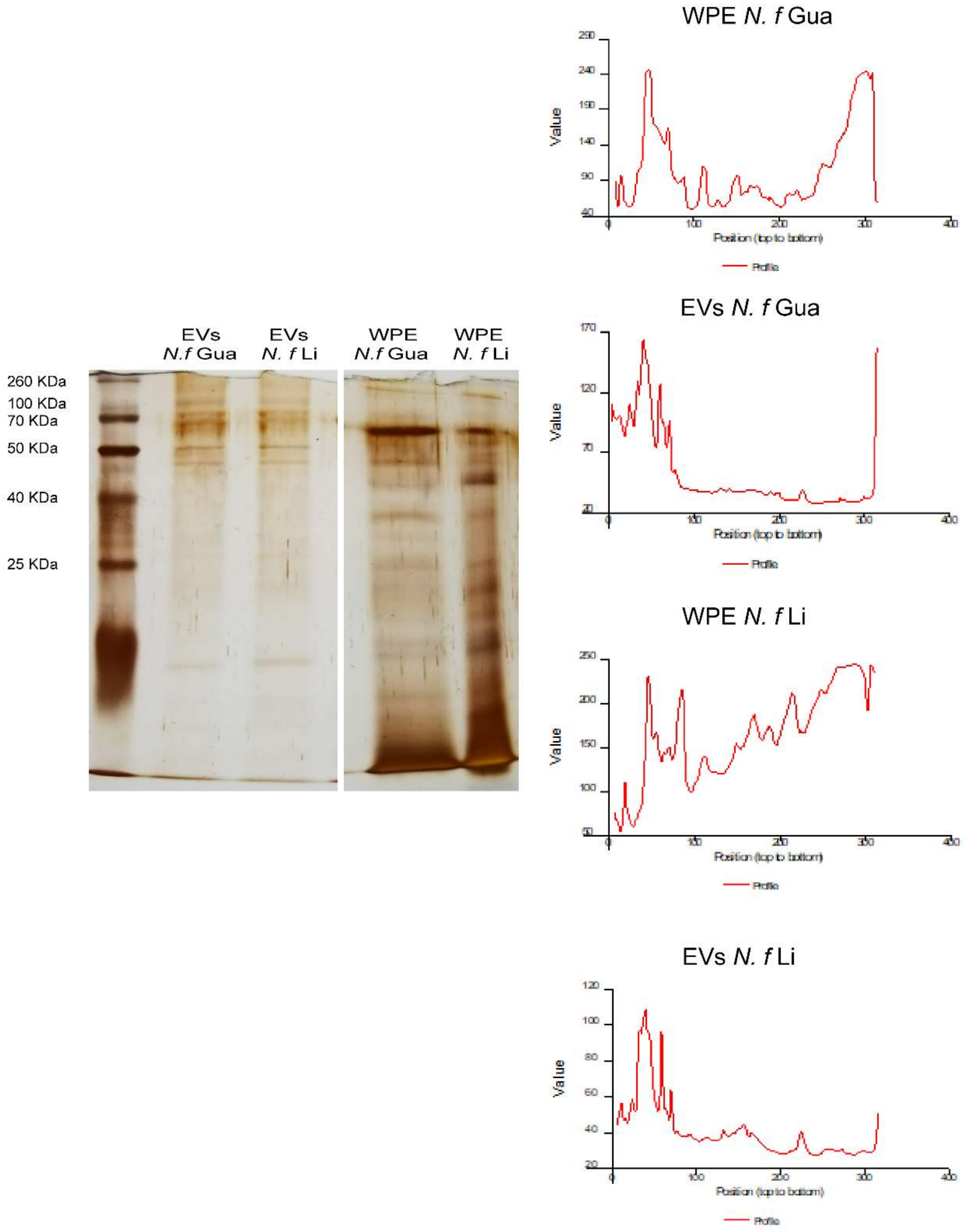

Figure S1. Protein profile and densitograms of extracellular vesicles secreted by *Naegleria fowleri* after SDS-PAGE electrophoresis and silver staining, in which similar band patterns in extracellular vesicles from isolates Guanacaste and Limón, which range from >15 kDa to 260 kDa, are presented. For this experiment, approximately 2 µg of protein/EV sample were loaded onto the gel and Spectra multicolor broad range protein ladder (Thermo Fisher Scientific, Waltham, Massachusetts, USA) was employed.

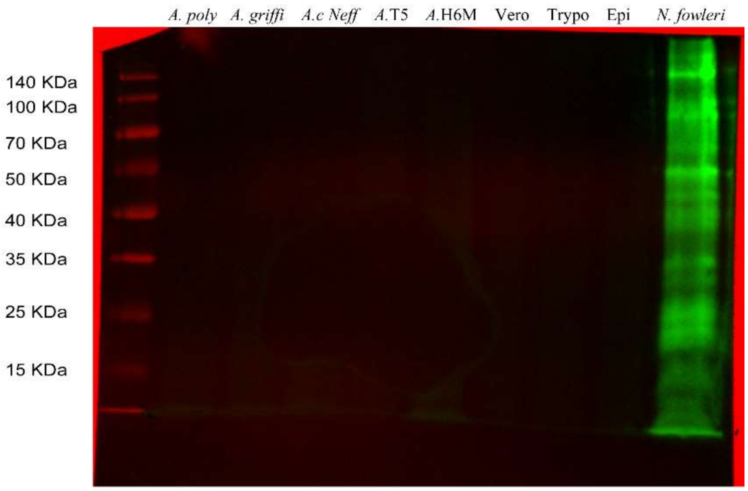

Figure S2. Western blot analysis that confirms the recognition of proteins in a lysate of trophozoites of *Naegleria fowleri* (ATCC *N. fowleri* Carter 30808). The recognition of proteins of other sources, including different genotypes of *Acanthamoeba* (*Acanthamoeba polyphaga, Acanthamoeba griffini, Acanthamoeba castellanii* Neff, *Acanthamoeba* genotype T5, *Acanthamoeba* H6M), different stages of *Trypanosoma cruzi* (trypomastigotes and epimastigotes) and the Vero cell line was also analyzed.

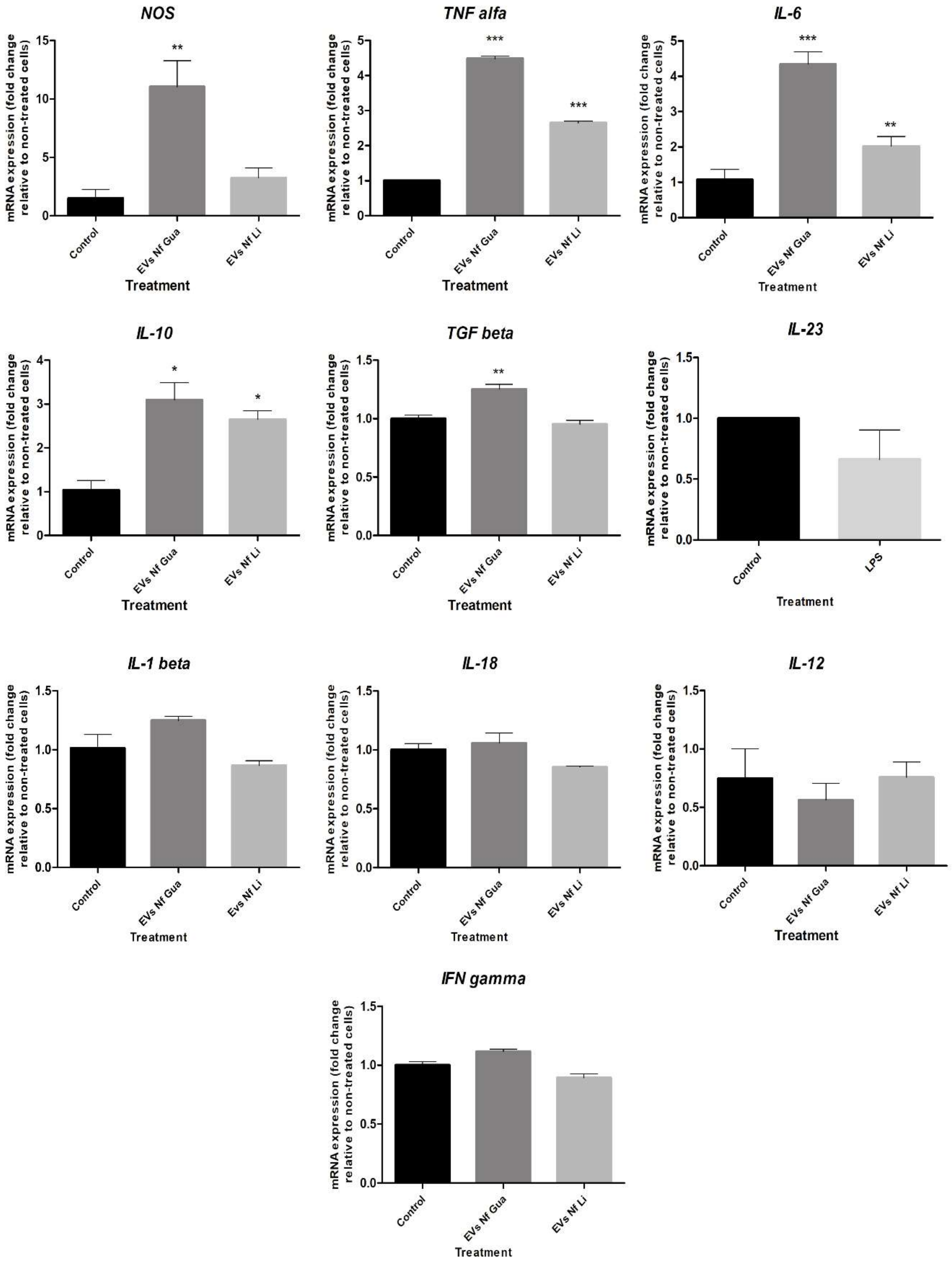

Figure S3. Differential mRNA expression analyses of *NOS* and cytokines after the incubation of primary cultures of mouse brain microglia with LPS. LPS was employed as the control of stimulation for expression analyses of primary cultures of mouse brain microglia with extracellular vesicles secreted *by Naegleria fowleri* Guanacaste and *N. fowleri* Limón. Values

are presented as the mean ± SD and one-way ANOVA with Tuckey post hoc test was performed for multiple comparisons to the negative control without treatment. *** $p < 0.0005$, ** $p < 0.005$.

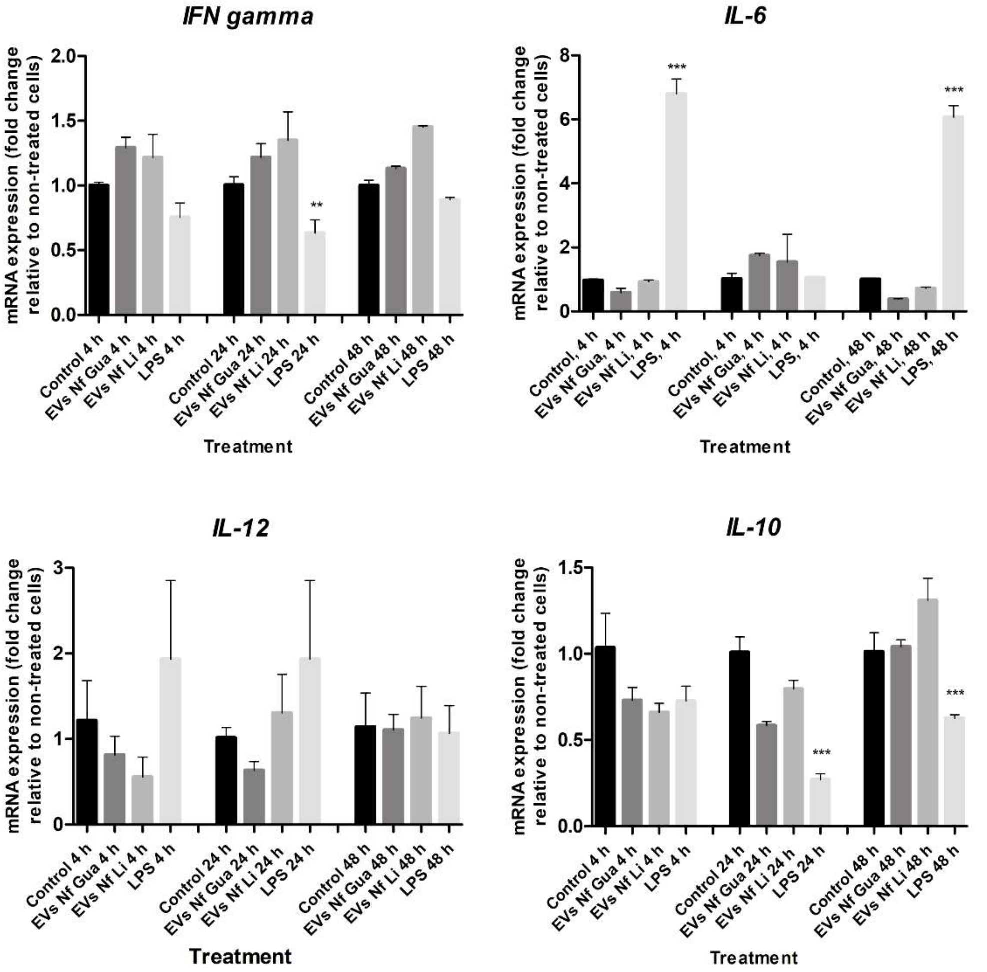

Figure S4. Differential mRNA expression analyses of cytokines after the incubation of BV2 cells with extracellular vesicles secreted *by Naegleria fowleri* Guanacaste and *N. fowleri* Limón. BV2 microglial cells were stimulated with extracellular vesicles (25 µg) of two clinic isolates of *N. fowleri* for 4, 24 and 48 hours and RTq-PCR was performed to analyze the expression pattern of cytokines. Values are presented as the mean ± SD and one-way ANOVA with Tuckey post hoc test was performed for multiple comparisons to the negative control without treatment. *** $p < 0.0005$, ** $p < 0.005$.
